# Stability Analysis of Metal Active-Gas Welding Short-Circuiting Transfer Based on Input Pulsating Energy

**DOI:** 10.3390/ma17020274

**Published:** 2024-01-05

**Authors:** Xiaoqing Lv, Quanjun He, Lianyong Xu

**Affiliations:** 1 School of Materials Science and Engineering, Tianjin University, Tianjin 300350, China; 2 Tianjin Key Laboratory of Advanced Joining Technology, Tianjin 300350, China

**Keywords:** short-circuiting transfer, input pulsating energy interpolation line, spectral analysis, approximate entropy

## Abstract

In this study, a platform for a welding experiment, used to collect input and output electrical signals, was constructed, and the algorithm for the input pulsating energy interpolation line (IPEI) was given. Experiments with MAG surface straight line welding were conducted at various voltages. Analysis of the IPEI in relation to the welding current was performed while combining real-world welding occurrences with high-speed camera images of droplet transfer. It was established that the IPEI can be employed as a characteristic parameter to assess the stability of the short-circuiting transfer process in MAG welding. The three criteria for assessing the stability were the spectrum, approximation entropy, and coefficient of variation. A comparative analysis was conducted on each of these approaches. It was determined that the most effective technique is approximation entropy. The approximation entropy of the welding current and IPEI are also highly consistent, with a correlation coefficient as high as 0.9889.

## 1. Introduction

MAG welding is widely used in the petrochemical, shipbuilding, engineering machinery, and automotive industries due to its high welding efficiency, good welding quality, stable arc, low spatter, strong penetration, and ease of automation [1]. The gathering and processing of the product manufacturing process and quality information has grown in importance as a component of information technology in the welding manufacturing business because of the rise in demand for high-quality, efficient, and refined welded goods [2]. The automation process of MAG welding is based on the monitoring of the stability of the welding process, which can be characterized by a variety of signals. The signals that can be collected during the welding process include electrical signals, acoustic signals, and high-speed photography, among which electrical signals are most commonly used due to their simplicity, reliability, and low cost [3].

Previous studies have searched for the welding electrical signal from a variety of perspectives to determine the stability of the welding process. Time domain statistical analysis is the simplest and most commonly used analytical method. Researchers have examined statistical characteristic parameters such as the arc duration, short circuiting time, and coefficient of the fluctuation of voltage and current to assess the stability of the welding process [4]. Feng [5] showed that it is feasible and practical to employ Mahalanobis distance, which is based on welding voltage and current, for a quality assessment of welding flaws in gas metal arc welding. Luksa [6] discovered a relationship between welding flaws and the mean and variance of momentary arc power. Spectral methods can analyze the distribution of the individual frequency components of the welding process. Suban [7] compared the welding current spectra under three different shielding gases and low and high welding parameters to determine the optimal shielding gas composition and welding parameters. Zhang [8] proposed a new algorithm based on the voltage power spectral density (PSD) calculated by the improved Welch’s algorithm for analyzing the arc voltage signal. Benaouda [9] used the single-sided amplitude spectrum (SSAS) and the power spectral density (PSD) to process the welding current to estimate the droplet transfer frequency under different droplet transfer modes and determined that the results obtained using PSD were more accurate. A welding electrical signal is a nonsmooth signal. Time–frequency analysis is one of the best and most accessible analytic techniques for nonsmooth signal processing [10]. Kaur [11] used a two-slit probe measuring device to measure the beam current, which was then processed by Discrete Wavelet Transformation (DWT). The wavelet coefficients were used to derive the energy distribution among different decomposition levels, which can characterize the electron beam better. Liu [12] employed ensemble empirical mode decomposition (EEMD) to obtain the frequency characteristics of the plasma plume morphology and built a support vector machine (SVM) model in combination with the raw signals in the time domain to classify the different penetration states. He [13] obtained the time–frequency distribution of the welding current signal by local mean decomposition (LMD) and Hilbert transformation, and evaluated the arc stability and welding formation quality by calculating the approximate entropy (ApEn) of the time–frequency distribution of the welding current signal. The welding electrical signal is also a nonlinear signal with chaotic and fractal characteristics. Therefore, the nonlinear dynamics method has a wide range of applications in the analysis of welding electrical signals. Gong [14] developed an approximate entropy (ApEn) model in order to quantify the effect of welding parameters on the process stability of narrow gap laser arc composite welding (NGHW). Gu [15] calculated the maximum Lyapunov exponent of characteristic currents during the welding process at different wire–wire distances using the phase space reconstruction technique and evaluated the stability of the welding process. Huang [16] introduced the multi-scale entropy method to analyze the current signals under different welding process parameters.

The parameters they selected were based on the output side, such as arc voltage and welding current, but the analysis of the input electrical signal of the welding machine was rarely covered. That was until Xiaoqing Lv and other researchers explored the connection between the input and output electrical signals of carbon dioxide arc welding and found that the short-circuiting transfer frequency of carbon dioxide arc welding calculated from the input pulsation energy and welding current maintains a consistent variation law [17].

A solid theoretical foundation exists for assessing the input side stability of the welding process. From the conservation of energy, it follows that the energy of the welding machine is supplied by the input terminal, and therefore, there must be a close interrelationship between the input and output terminal. The more stable a welding process is, the better the cyclicity of the droplet transfer. It means that the welder’s input energy will be characterized by regular cycles. The more unstable the welding process is, the less cyclical the droplet transfer is and the more volatile the signal at the input is.

Judging the stability of the welding process from the input side has its unique advantages. Firstly, gathering input signals is considerably simpler compared to gathering electrical output signals. The similar input power levels of different types of welders facilitate hardware modularization. For different types of welding machines from different equipment manufacturers, the same hardware can be used for the input evaluation, and only the evaluation algorithm needs to be modified. Secondly, the energy management of the welding apparatus is made easier by the analysis of the incoming electrical data. The input electrical signals make it easier to monitor the energy consumption of the welding machine as a way to determine the actual energy requirements of the equipment and system. Lastly, welder input-based control systems aid in lowering the complexity and expense of welding production automation. They also offer fresh concepts for the predictive maintenance of the welding production workshop and encourages the conversion of the welding production workshop to the group control management mode of the welding machines. Therefore, studying the electrical signal input of the welding machine has practical significance.

The proper analysis technique and characteristics of the welding machine’s input electric signal are chosen for this research. The purpose is to investigate the stability performance characteristics of MAG welding short-circuiting transfers at different given voltages, and then to determine the appropriate input characteristic parameters and stability assessment methods.

## 2. Experimental Methodology and Analysis of Results

### 2.1. The Experimental Platform, Materials, and Methods

**Figure 1:**
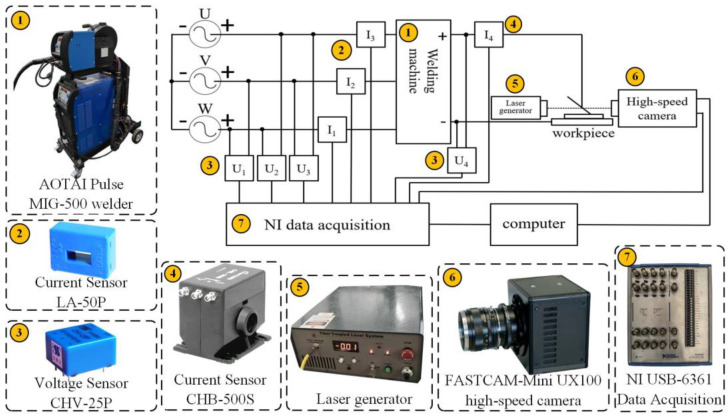
Schematic diagram of the experimental platform.

The experimental platform, shown in Figure 1, includes a welding test system consisting of a Pulse MIG-500 welder (AOTAI Shandong, Jinan, China), a walking mechanism, and a data acquisition system consisting of an NI data acquisition, a FASTCAM-Mini UX100 high-speed camera (Photron Yamagata, Tokyo, Japan), a computer, voltage sensors, and current sensors.

**Figure 2:**
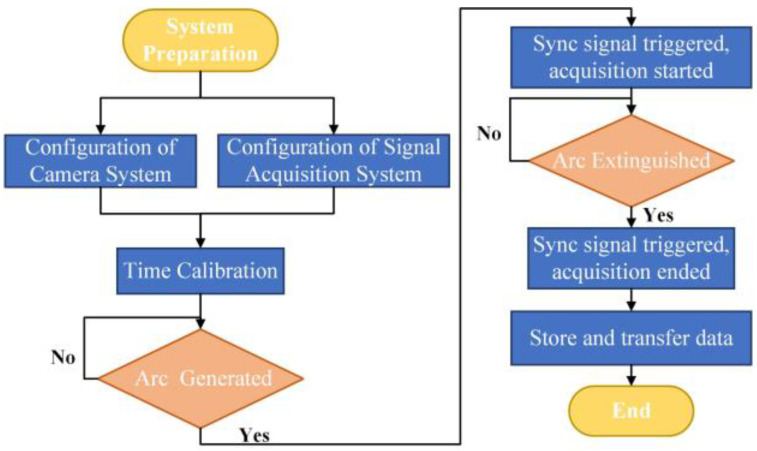
Flow diagram for the synchronizing high-speed camera and electrical signal acquisition.

The signal acquisition flow is shown in Figure 2. Firstly, configure the high-speed camera system and electrical signal acquisition system. After the arc is generated, the synchronization signal is triggered, initiating the high-speed camera and electrical signal acquisition. Both the electrical signal collection and camera recording cease when the arc is extinguished. Then the high-speed camera pictures and electrical signals are stored, and the next round of experiments is performed.

The welded workpiece is Q235 mild steel with a plate thickness of 5 mm, the wire grade is THQ-50C with a diameter of 1.2 mm, and the shielding gas is a mixture (Ar 82% + CO_2_ 18%). The experimental parameters are as follows: the distance from the contact tube to the workpiece is 13 mm, the welding speed is 30 cm/min, and the flow rate of the shielding gas is 15 L/min. The data acquisition system has a sample frequency of 12,000 Hz and collects 10 s of data for each welding seam.

At a given wire feed rate of 5 m/min, the given voltage is uniformly ramped from 15 V to 28 V (at 1 V intervals) for the flat lay welding experiment. The current and voltage data are collected for the input and output sides.

### 2.2. Experimental Results

The actual arc voltage, welding current, welding phenomena, and weld seam formation at different given voltages are shown in Table 1. The arc voltage and welding current are averages obtained from the data acquisition card. As can be seen from the phenomena in Table 1, the voltage gradually increases, and the stability of the welding process changes from unstable to stable and back to unstable. The stability is divided into four simple cases, identified by the different background colors in Table 1.

It can be seen that at a given voltage of 15/16 V, the measured arc voltage differs greatly from the given voltage and the welding current is also smaller. This is because a continuous and stable arc cannot be formed due to the mismatch of welding parameters. During the extinguishing of the arc, the welding current is close to 0, while the voltage is high until the arc burns again. As a result, as shown in Table 1, the mean values of the arc voltage and welding current are displaced at a given voltage of 15/16 V.

A more thorough analysis of the experimental data is conducted later. The analysis is conducted using high-speed photographic pictures of the melt drop transition and electrical signals at the inputs and outputs.

## 3. Input Electrical Signal Processing

**Figure 3:**
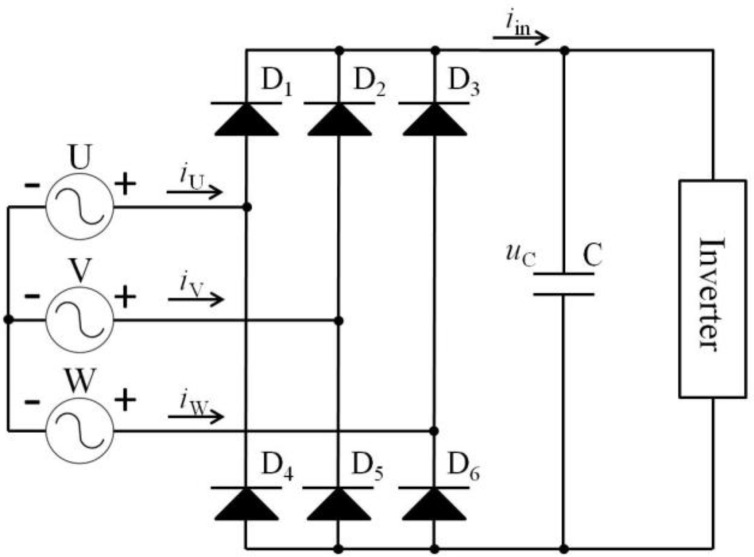
Three-phase full-bridge uncontrolled rectifier circuit.

The preferred power supply strategy for contemporary arc welding machines is an inverting topology with a three-phase unregulated rectifier (as indicated in Figure 3).

**Figure 4:**
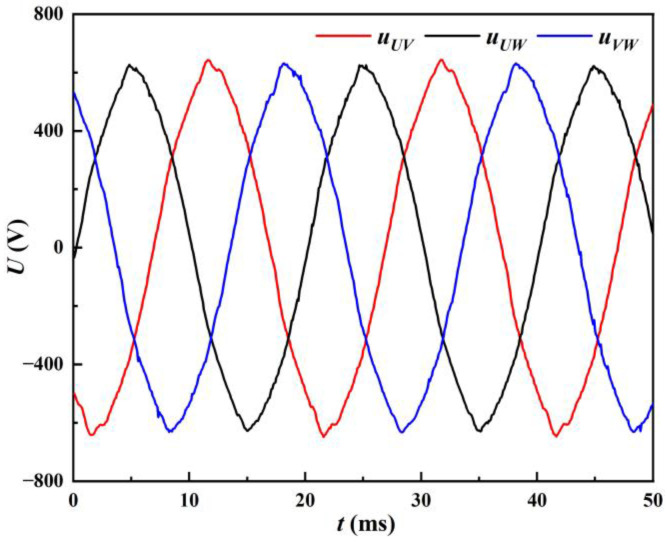
Input line voltage of the welding machine.

**Figure 5:**
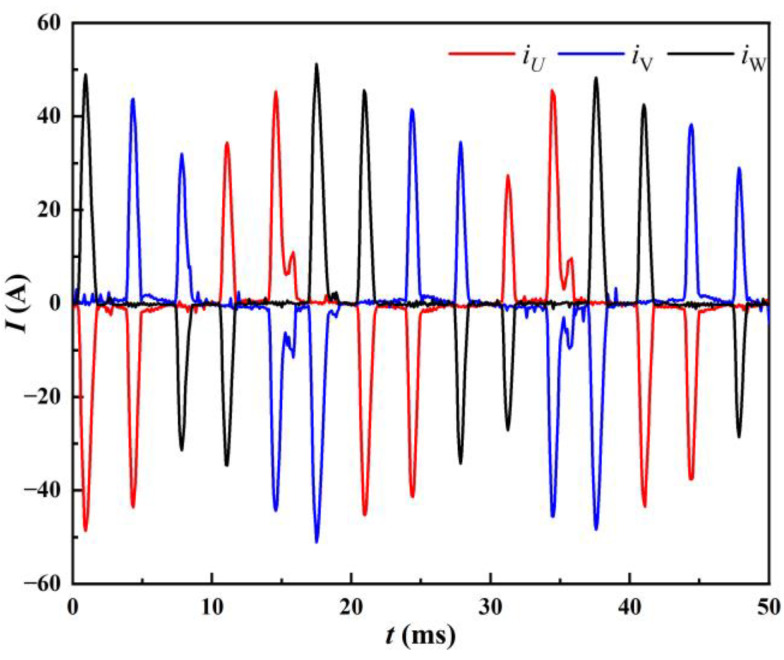
Input line current of welding machine.

The parameters that can be directly collected at the input side of the welding machine are the input three-phase line voltages *u*_UV_, *u*_UW_, *u*_VW_ and the input three-phase line currents *i*_U_, *i*_V_, *i*_W_, whose waveforms are shown in Figure 4 and Figure 5, respectively. *u*_UW_ is the line voltage between the U and W phase. Similarly, u_VW_ corresponds to the V and W phase, and *u*_UV_ corresponds to the U and V phase. *i*_U_, *i*_V_, and *i*_W_ correspond to the U, V, and W phases, respectively. During the welding process, when the maximum absolute value of the three-phase line voltage is greater than the capacitor C voltage, the input side provides energy to the arc load while charging the capacitor C, *i*_in_ > 0. When the maximum absolute value of the three-phase line voltage is less than the capacitor C voltage, the capacitor C provides energy to the arc load, *i*_in_ = 0. For example: In Figure 4, around 5 ms, *u*_UW_ reaches its peak. The input side runs simultaneously with the capacitor C and the arc load power supply, at this time diode D1, D6 conduction; that is, *i*_U_ = *i*_in_, *i*_W_ = −*i*_in_, *i*_V_ = 0, corresponding to the current wave at about 5 ms in Figure 5.

Therefore, the instantaneous input power *P*_in_ is calculated from the input voltage and input current [17] as follows.

(1) $$P_{in}=u_{in}\times i_{in}$$

Since the input three-phase line current is symmetrically distributed on the *x*-axis, *i*_in_ is the positive part of the three-phase line current. *u*_in_ is the absolute maximum value of the three-phase line voltage.

(2) $$u_{in}=\max(\left|u_{UV}\right|,\left|u_{UW}\right|,\left|u_{VW}\right|)$$

**Figure 6:**
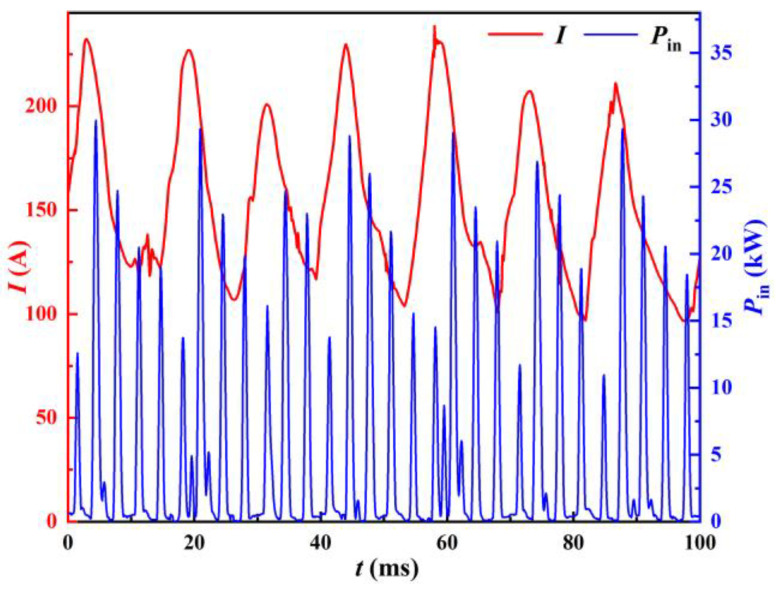
Instantaneous input power and welding current.

**Figure 7:**
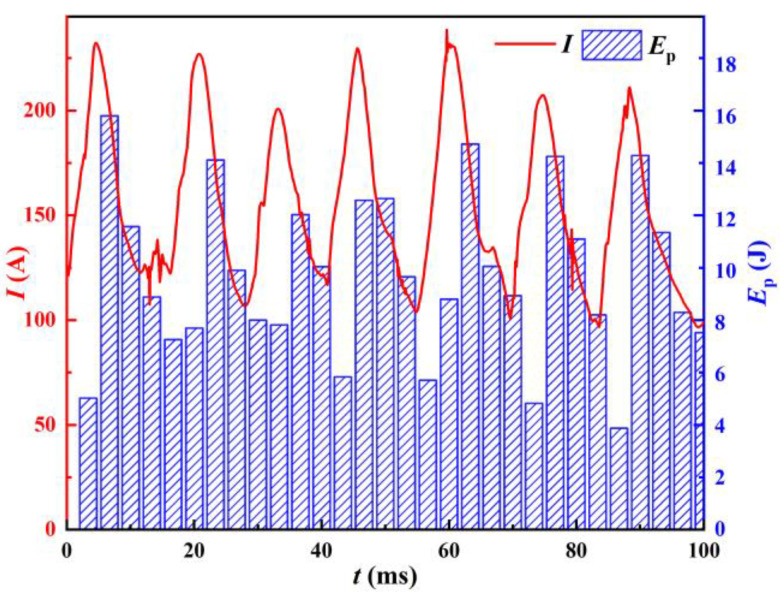
Input pulsating energy and welding current.

The instantaneous input power *P*_in_ versus the welding current is shown in Figure 6. Power is supplied to the welder in the form of pulsating peaks. A calculation of the input energy during each pulsation cycle T gives the input pulsation energy *E*_P_, which corresponds to the welding current as shown in Figure 7.

Combined with Figure 6 and Figure 7, it can be seen that the peaks of the instantaneous input power and the input pulsating energy both have a consistent trend with the weld current and have a certain hysteresis. This is because the change in the welding current during the welding process is continuous, while the energy input does not change continuously. When the instantaneous input power is 0, the capacitor C in the circuit provides energy for welding, causing the instantaneous input power and input pulsating energy changes to lag behind the welding current. As can be seen in Figure 6, there are minor oscillations near the base of the power wave (e.g., at 60 ms in Figure 6). These fluctuations often occur when the input power is high and also provide energy for the welding process. If the peak of the input power is taken as the characteristic of the input side, this part of the input energy is neglected. Therefore, the input pulsating energy is a better representation of the actual welding process.

**Figure 8:**
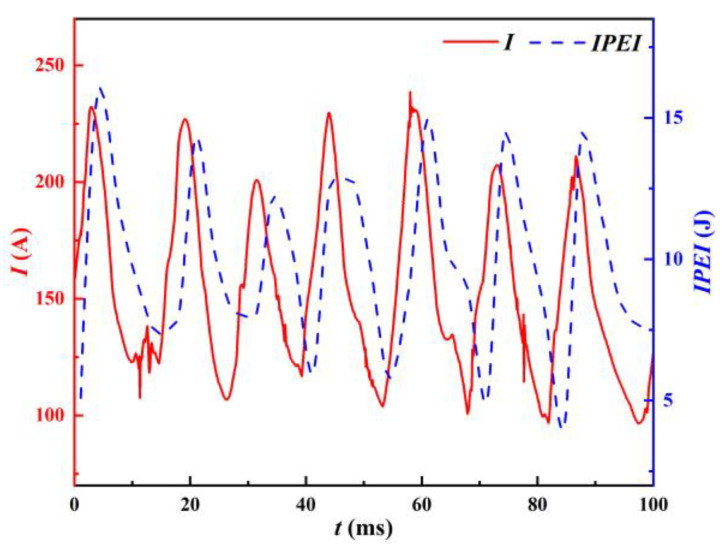
IPEI and welding current.

Both the instantaneous input power and pulsation energy fluctuation frequency are 300 Hz. Although the input pulsating energy can be a good representation of the welding current variation trend, obviously, the quantity of data is relatively small when analyzing the stability characteristics. To facilitate comparison with the output electrical signal, the input pulsed energy is linearly interpolated with reference to the sampling frequency of 12,000 Hz at the output side. That is, 39 data are interpolated between every two data points, thus ensuring a consistent amount of the input and output signal data. The input pulsating energy interpolation line is referred to as the IPEI to simplify the description. The IPEI of the welding machine and the welding current are compared in Figure 8, which demonstrates that the two trends are essentially the same.

Due to the IPEI and welding current’s uniformity, weld shaping and high-speed camera images of the molten drop transition phase were combined with the experimental data for a thorough analysis.

## 4. Analysis of Experimental Results and Presentation in Electrical Signals

**Figure 9:**
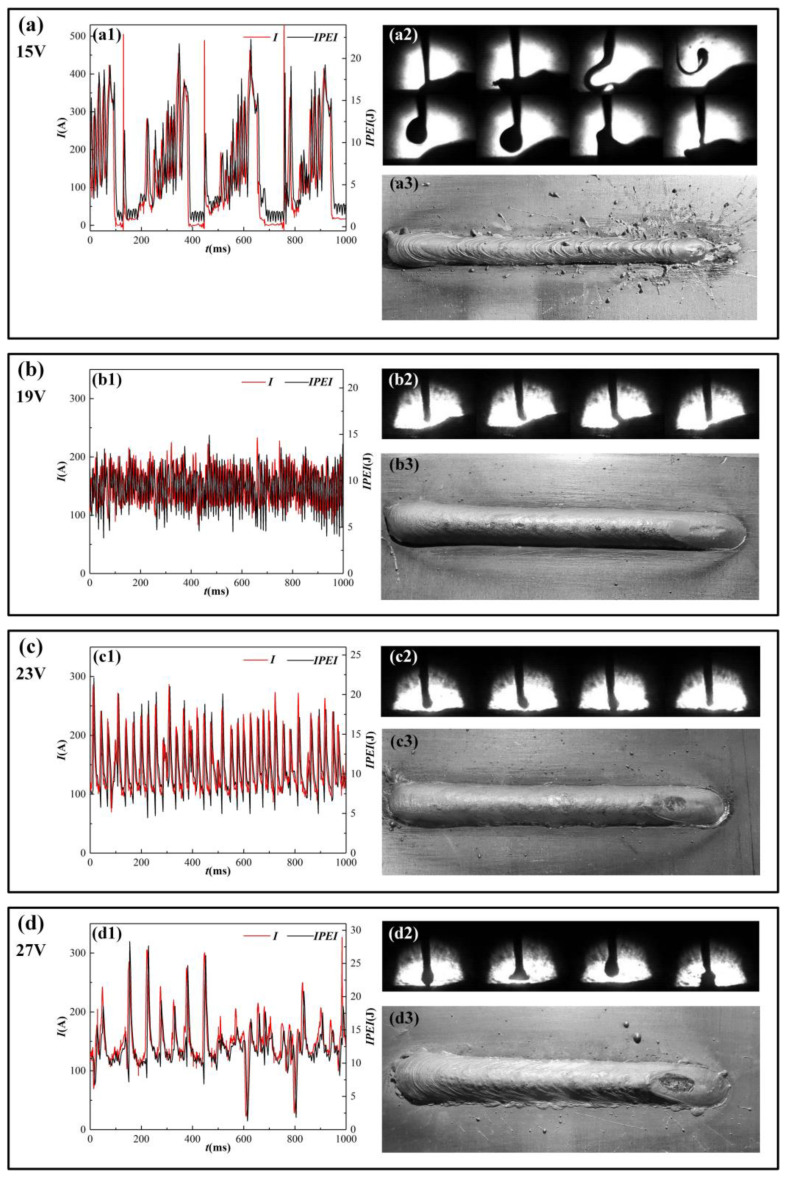
Welding current and IPEI waveforms, high-speed camera photos, and weld seam formation at different given voltages (**a**: **a1**–**a3**) 15 V, (**b**: **b1**–**b3**) 19 V, (**c**: **c1**–**c3**) 23 V, (**d**: **d1**–**d3**) 27 V.

Due to the IPEI and welding current’s uniformity, weld shaping and high-speed camera images of the molten drop transition phase were combined with the experimental data for a thorough analysis. In combination with the welding phenomena and weld formation in Table 1, the representative given voltages were selected (15 V, 19 V, 23 V, and 27 V), as shown in Figure 9.

Mutual verification with electrical signals and a more intelligible display of the short-circuit changeover process are made possible by high-speed camera images [18]. The MAG welding short-circuiting transfer stability is analyzed in combination with the above information. When the given voltage is between 15 V and 17 V, as in Figure 9a, the mismatch between the given voltage and the wire feed rate leads to difficulty in starting the arc and prevents stable welding. The extended growing process and the vast size of the droplet are shown in the high-speed camera image of the molten droplet. The lower portion of Figure 9(a2) illustrates the aforementioned process, which correlates to the droplet growth process at 800 ms in the waveform graph of Figure 9(a1). When compared to other parameters, it is evident that the droplet growth is more pronounced. When the big drops fall into the molten pool, they induce significant vibrations that cause spatter. Throughout the welding process, the droplet size and transition frequency are not constant. The extension of the welding wire into the molten pool is a frequent occurrence and results in an extremely long short circuit period and an arc explosion. The upper portion of Figure 9(a2) illustrates the aforementioned process, which correlates to the wire reaching into the molten pool at 450 ms in the waveform graph of Figure 9(a1). It is evident that the wire extends straight into the molten pool, where it splits and deforms while the molten pool fiercely vibrates [19]. The waveforms of the IPEI and welding current are both very erratic. The given voltage and the actual arc voltage are very different. The welding current and IPEI waveforms both exhibit this droplet transition behavior. In other words, the waveform’s various peaks and the uneven space between them are reflections of the heterogeneity in droplet size and transition frequency. According to Figure 9b, when the provided voltage is applied into the stable welding interval (18–20 V), there is no evident growing process of the uniformly sized droplet, which is a little smaller than the wire diameter, shown in Figure 9(b2). The short-circuiting transfer frequency is high and the molten pool vibration is low. The weld seam is well formed with minimal spatter. At this stage, the IPEI and welding current peaks have low peak variations. The waves have uniformly and minutely spaced peaks. As the given voltage exceeds 21 V, the droplet volume increases as the voltage does, and the frequency of the short-circuiting transfer steadily reduces. Weld formation is still good. However, when the voltage rises, spattering starts to happen and becomes worse. The waveform in Figure 9c demonstrates that the current undulation increases and the short-circuiting transfer frequency decreases for a given voltage of 23 V in comparison to a given voltage of 19 V. However, when the given voltage is greater than 25 V, as shown in Figure 9d, the droplet grows obviously, the shape is not uniform, and it keeps spattering in the process of growing. In addition, during the growth of the droplet, it is often in transient contact with the melt pool and then thrown away, causing a large spatter, as shown in Figure 9(b2). The amount of spatter and its individual particles are growing. The arc sound starts to be garbled. Short-circuiting transfer frequency continues to decline, and the uniformity of the short-circuiting transfer process decreases. The peak of the welding current and IPEI vary considerably during the short-circuiting transfer. As can be observed, similar to the welding current, the IPEI offers a useful illustration of the short-circuiting transfer process at various voltages. This allows for the observation of details such as the frequency of the short-circuiting transfer and the length of the molten droplet. It is shown that IPEI and welding current are closely related. Therefore, the IPEI can be used to assess the stability of the short-circuit changeover process. The specific analysis method is investigated in the following section.

The IPEI is analyzed by the spectrum, coefficient of variation, and approximate entropy and compared with the conclusions of the current analysis in order to show that it is feasible to use the IPEI to evaluate the stability of the short-circuiting transfer process in MAG welding. The analysis method includes considerations from the time domain, frequency domain, and nonlinear dynamics perspectives. The most popular technique for evaluating the stability of the welding process is time domain analysis. Analysis of the distribution of various frequency components in the welding electrical signal is possible using the frequency domain perspective. Additionally, since welding is a nonlinear process by definition, the use of nonlinear approaches to evaluate stability has grown during the past ten years.

## 5. Spectrum Analysis of IPEI

Spectral analysis is a common method for analyzing welding electrical signals. For the short-circuiting transfer process, the spectrum of the welding current can visualize the short-circuiting transfer frequency. Analyzing the spectrum of the IPEI and welding current can be used to verify the validity of the IPEI assessment of the welding process from a frequency domain perspective. The method of obtaining the spectrum is the discrete Fourier transform, with Equation (3):

(3) $$F_{N}\left(\omega \right)=\sum_{n=0}^{N-1}X(n)e^{-j\omega n}$$

where X(n) is the acquired time-domain signal; and $\omega =\frac{n}{NT}\;,\;(n=0,\;1,\;2,\;\ldots ,\;N-1)$.

**Figure 10:**
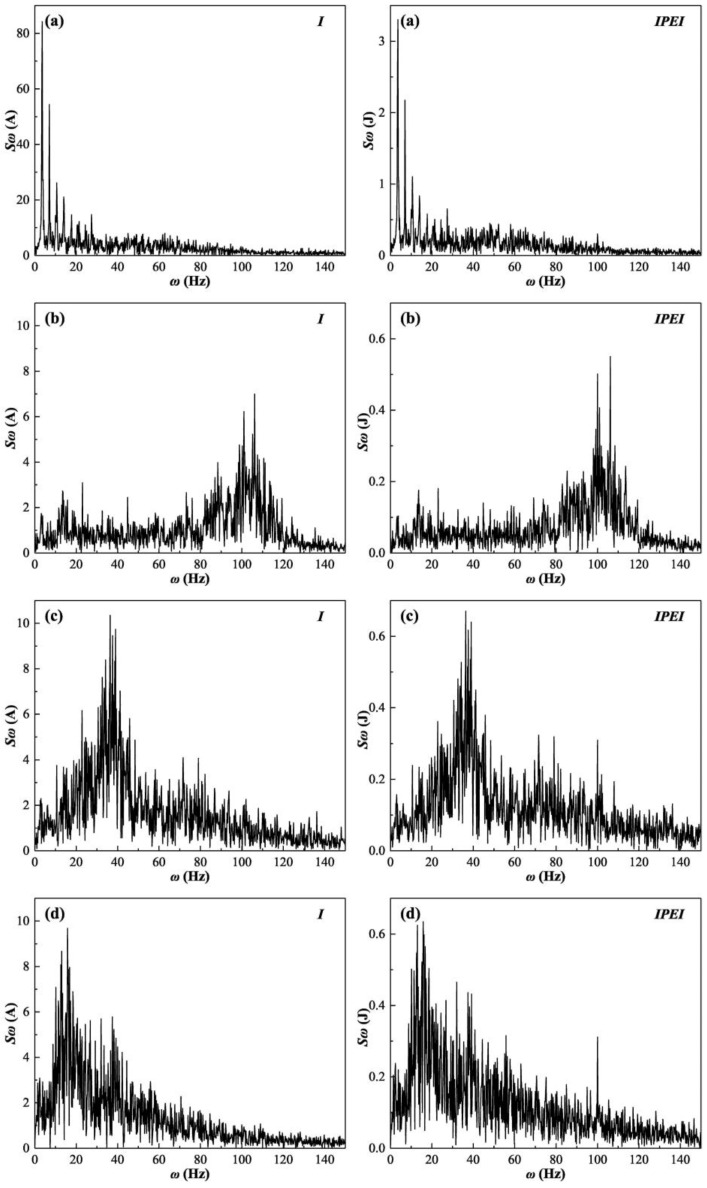
Spectra of welding current and IPEI at different given voltages. (**a**) 15 V, (**b**) 19 V, (**c**) 23 V, (**d**) 27 V.

For the IPEI and welding current at given voltages of 15 V, 19 V, 23 V, and 27 V, the discrete Fourier transform (DFT) is performed to obtain the spectra. A comparative analysis of the frequency spectra of the two is performed. The spectra of the IPEI and welding current are shown in Figure 10.

The four given voltages used to plot the spectra are divided into four different cases in Table 1. Comparing the spectra of the welding current and IPEI, it can be seen that the spectra of both are basically the same during the welding process with different stability. Not only is there a similarity in the overall trend, but there is also a good connection in specific distinctive wave peaks. The frequency corresponding to the largest amplitude in the spectrum is called its dominant frequency. It can be found that the dominant frequencies in the spectrum plots of the welding current and IPEI for the same parameters are identical. Comparing the wave peaks in the spectrum plots of the welding current and IPEI, it can be seen that the composition of the different frequency components of both is basically the same. It can be concluded that the IPEI not only provides a good fit of the welding current in the time domain but also retains most of the characteristics of the welding current in the frequency domain.

In the frequency domain, the stability of the MAG welding short-circuiting transfer procedure can also be evaluated. At a given voltage of 15 V, the welding process does not proceed steadily. The short-circuiting transfer interval varies considerably and it is accompanied by wire burst. The spectrum of the welding current and IPEI shows multiple relatively independent peaks at lower frequencies (0–30 Hz). As the given voltage increases to 19 V, the welding process becomes stable and the short-circuiting transfer frequency increases. Both the welding current and IPEI show that the dominant frequency is between 100 Hz and 110 Hz, which is exactly the same as the transition frequency shown on the time domain in Figure 10b. Similarly, at voltages of 23 V and 27 V, the dominant frequencies in the corresponding spectra are consistent with the short-circuiting transfer frequencies shown in the time domain of Figure 10c,d. In general, the welding process is more stable if the short-circuiting transfer frequency is higher and its distribution is more concentrated. Since the spectrum plot can clearly obtain the transition frequency situation [9], the stability can also be effectively observed from the frequency domain of the IPEI.

The study based on the spectrum, however, lacks sufficient intuition and cannot be quantified. Furthermore, this method requires a certain length of time, which makes it difficult to track variations in the welding process’s stability over time. Therefore, the coefficient of variation and approximate entropy were also selected in order to identify the feasibility of assessing the stability of the welding process through the IPEI from the perspective of the time domain and nonlinear dynamics.

## 6. Coefficient of Variation and Approximate Entropy of IPEI

The coefficient of variation (CV) is an important indicator of data dispersion and is defined as the ratio of the standard deviation to the mean, as shown in Equation (4).

(4) $$CV\left(x\right)=\frac{std(x)}{mean(x)}$$

The CV is an important parameter in the time domain analysis of welding electrical signals. A higher CV indicates a higher degree of data dispersion, which means that the waveform is more volatile. When it is reflected in the welding current, it means that the welding process is less stable.

The approximate entropy (ApEn) algorithm was proposed by Steve Pincus in 1994 as a physical quantity to describe the complexity of a time series. The ApEn gives stable quantization results for nonsmooth, nonlinear sequences, so it can be applied to the analysis of welding electrical signals. ApEn is calculated as follows [20]:

The time series *u*(*i*) with data length N is reconstructed in m-dimensional phase space, and the reconstructed vector sequence is shown in Equation (5):

(5) $$X_{i}^{m}=\left\{u\left(i\right),u\left(i+1\right),\ldots ,u\left(i+m-1\right)\right\}\;\;\;\;\;\;\;(\;i=1,\ldots ,N-m+1)$$

where *m* is the length of the data segment used as the approximate comparison.

The distance $d_{ij}^{m}$ between the series $X_{i}^{m}$ and $X_{j}^{m}$ is defined as the maximum value of the difference between their corresponding endpoints, as follows:

(6) $$d_{ij}^{m}=d\left[X_{i}^{m},X_{j}^{m}\right]=\underset{k\in (0,m-1)}{\max}\left|\left(u\left(i+k\right)\right)-\left(u\left(j+k\right)\right)\right|$$

The Heaviside function is introduced, which makes a judgement on whether a data segment is approximate or not:

(7) $$H\left(r{-d}_{ij}^{m}\right)=\left\{\begin{array}{c} 0,\;\;\;r{-d}_{ij}^{m}<0\\ 1,\;\;\;\;r{-d}_{ij}^{m}\geq 0 \end{array}\right.$$

where *r* represents the width of the boundary of the Heaviside function; if the distance between the series is less than the r, the two sequences can be considered approximate.

$C_{k}^{m}(r)$ is defined as the ratio of the number of approximate series to the number of all series *N* – *m* + 1, known as the probability that the two series are approximate, as is shown in Equation (8):

(8) $$C_{k}^{m}\left(r\right)={\left(N-m+1\right)}^{-1}\sum_{k=1}^{N-m+1}H_{k}\left(r{-d}_{ij}^{m}\right)$$

Take the logarithm of $C_{k}^{m}\left(r\right)$ and then find its mean for all k and define the function $\phi^{m}$ as follows:

(9) $$\phi^{m}\left(m,r\right)={\left(N-m+1\right)}^{-1}\sum_{k=1}^{N-m+1}lnC_{k}^{m}\left(r\right)$$

Similarly, making *m* = *m* + 1 and repeating the above calculation yields $\phi^{m+1}$.

Finally, the approximate entropy is defined as the difference between $\phi^{m}$ and $\phi^{m+1}$, as shown in Equation (10):

(10) $$ApEn\left(m,r\right)=\phi^{m}\left(m,r\right)-\phi^{m+1}\left(m,r\right)$$

Generally, in stability analysis, the mode dimension *m* = 2 and the allowable deviation *r* is taken as 0.1–0.25 times the standard deviation of the series; here, r is taken as 0.15 [20].

The collected welding current and IPEI data were divided into 250 ms long groups with 3000 data each. Each group of data’s CV and ApEn were separately computed, along with their mean values, and the findings are displayed in Table 2.

**Figure 11:**
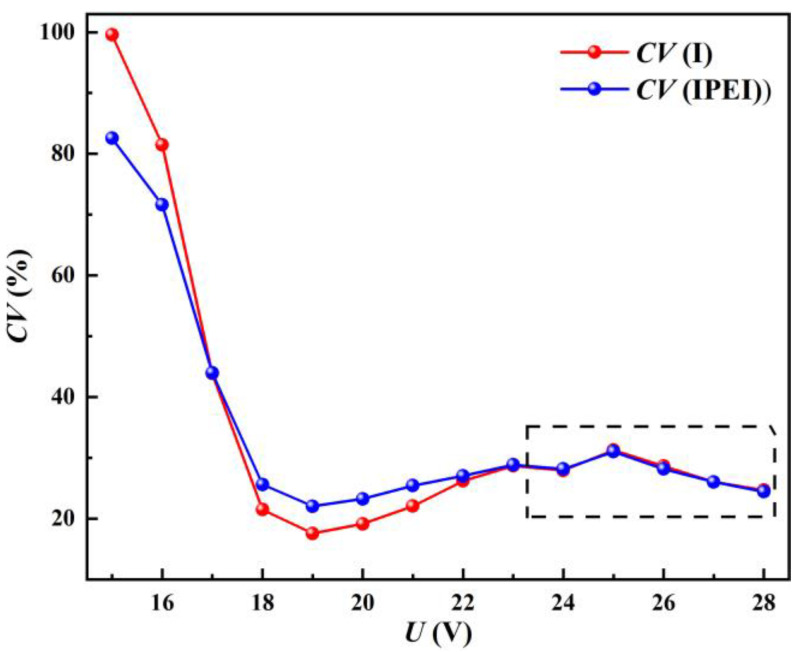
The comparison of the CVs for IPEI and welding current.

The CVs of the welding current and IPEI were compared with the welding phenomena, and it was found that the CVs of the two were highly consistent with a given voltage change, as in Figure 11. The CVs of the welding current and IPEI have a high correlation coefficient of 0.9987. The validity of the IPEI as an object of stability analysis is demonstrated from the time domain perspective.

At a given voltage of 15–17 V, stable welding is prevented. The weld current and IPEI waveforms were scattered with great fluctuation, and the CVs of both were also great at this time. As the given voltage enters the stable welding interval (18–20 V), the trends of the CVs of the welding current and IPEI are also at a minimum at 19 V. As the given voltage continues to grow, along with the generation of spatter, the stability of the welding process begins to decline. The CVs of the welding current and IPEI subsequently become larger.

However, even though the stability of the welding process continuously declines, the CVs of the welding current and IPEI do not continuously increase when the provided voltage is larger than 24 V. Instead, after the given voltage exceeds 25 V, the CV falls as the given voltage rises. The trend is marked in a square in Figure 11. It indicates that CV as a determination method is deficient at higher given voltages.

The stability decreases when the given voltage is greater than 24 V, but the CV decreases as well. The mathematical basis of the CV dictates this. This is due to the fact that as the given voltage rises, the frequency of short-circuiting transfers decreases and the interval between each short-circuiting transfer increases. The waveform between the two short-circuiting transfers is smoother, and close to the mean. As a result, it lowers the CV’s value and intensifies as the applied voltage rises. In the end, this impact cancels out the CV’s rise brought on by the welding process’ instability. Thus, the CVs of the welding current and IPEI show a decreasing trend.

**Figure 12:**
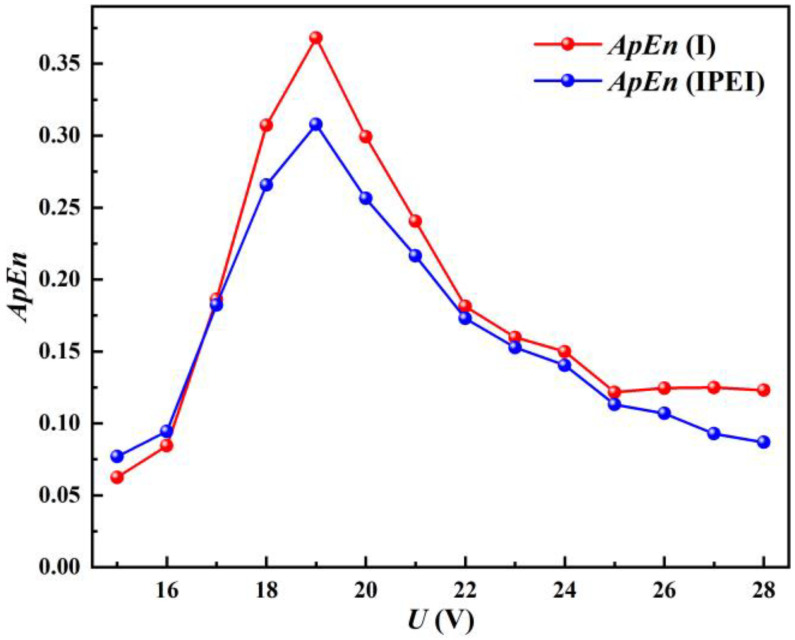
The comparison of the ApEns of IPEI and welding current.

Figure 12 gives the ApEn trends of the welding current and IPEI. The figure shows that the ApEns of the welding current and IPEI are also highly consistent, with a correlation coefficient as high as 0.9889.

For short-circuiting transfers, the higher the transition frequency, the more stable the welding process will generally be. A high transition frequency also means that the number of fluctuations per unit time is relatively more complex and the ApEn will be higher. This aspect has been confirmed in the references [21].

Through the former analysis, combined with Table 1 and Figure 12, it is shown that the welding process is extremely unstable at a given voltage of 15–17 V. The ApEns of both the welding current and the IPEI are quite minimal at this stage. As the given voltage increases, steady welding soon begins. The short-circuiting transfer frequency gradually increases, and the ApEns of the welding current and IPEI also increase. The ApEn reaches its maximum at 19 V, which is consistent with the CV calculation. As the given voltage continues to increase, the stability gradually starts to deteriorate. The ApEns of the welding current and IPEI also decrease. This shows that the IPEI can also characterize the welding process in a nonlinear perspective.

Additionally, it is discovered that ApEn successfully overcomes the CV’s shortcoming in evaluating stability. That is, at higher given voltages (greater than 23 V), as the voltage increases, the stability of the short-circuit process decreases and the CV criterion decreases instead. Because the ApEn characterizes the complexity of the time series, at higher voltages, the short-circuiting transfer frequency decreases as the stability becomes worse. The complexity of the welding current and IPEI decreases simultaneously. The ApEn of both gradually decreases. Therefore, the ApEn is more effective than the CV in assessing the stability of short-circuiting transfers.

## 7. Conclusions

The main findings of this paper are as follows:

(1) By comparing the input signals and welding current signals during the short-circuiting transfer of MAG welding, it is found that the peak instantaneous power and pulsating energy at the input side are in good agreement with the variation in the welding current. The input pulsating energy was interpolated, and the stability of the welding process was judged by this interpolated line from the time domain (CV) and frequency domain (spectrum), and from the nonlinear perspective (ApEn). It was found to be in perfect agreement with the trend of the welding current as the studied covariate. The feasibility of using the input pulsating energy interpolation line (IPEI) to gauge the stability of the welding process is proven.
(2) By comparing the stability of the short-circuiting transfer process at different given voltages, combined with the performance of the spectrum, ApEn, and CV as stability criteria, it is shown that all three can effectively assess the stability of the welding process. The CV and ApEn are more intuitive and quantitative when used as criteria. However, the CV acts in a manner that is inconsistent with the trend features of stability at voltages higher than 23 V. In contrast, ApEn behaves consistently, confirming that ApEn is better as a stability criterion.

A limitation of this study is that only the constant voltage mode short-circuiting transfer process is examined. An examination of the input electrical signals for various droplet transfer modes and welding techniques can come next. In the future, research will continue to improve the system of evaluating the stability of the welding process by the input electrical signal and provide fresh concepts for process monitoring in welding.

## Figures and Tables

**Table 1:** Experimental results at different given voltages.

Given Voltage *U*/V	Arc Voltage $\bar{U_{a}}/V$	Welding Current $\bar{I_{out}}/A$	Welding Phenomena and Weld Seam Formation
15	36.82	121.54	Unable to weld stably, the arc bursts, the spatter is extremely large, and the molding is extremely poor.
16	24.55	127.29	
17	17.63	143.51	
18	18.27	147.86	The stable arc; the spatter is quite small. The arc sound is regular; the welding seam is well formed.
19	19.19	149.76	
20	20.23	147.95	
21	21.20	148.64	A small amount of spatter is produced. The weld seam formation is relatively good.
22	22.28	145.93	
23	23.26	147.09	
24	24.22	149.68	
25	25.24	148.21	The number and size of spatters increase. Arc sound starts to be messy. The weld seam is poorly formed, with biting edges.
26	26.21	149.43	
27	28.92	150.17	
28	28.20	150.81	

**Table 2:** The CV and ApEn of welding current and IPEI.

Given Voltage *U*/V	CV (*I*_out_)/%	CV (IPEI)/%	ApEn (*I*_out_)	ApEn (IPEI)
15	99.58	80.58	0.0625	0.0717
16	81.46	69.40	0.0845	0.0892
17	43.86	41.44	0.1862	0.1788
18	21.51	23.15	0.3071	0.2651
19	17.59	19.35	0.3681	0.3171
20	19.17	20.93	0.2992	0.2593
21	22.07	23.41	0.2405	0.2113
22	26.24	25.44	0.1813	0.1630
23	28.71	27.33	0.1598	0.1428
24	27.95	26.77	0.1499	0.1320
25	31.28	29.76	0.1256	0.1054
26	28.65	27.04	0.1245	0.1023
27	26.06	25.14	0.1249	0.0902
28	24.72	23.62	0.1231	0.0844

## Data Availability

Data are contained within the article.

## References

[1] Kumaran T.A.V., Reddy S.A.N.J., Jerome S., Anbarasan N., Arivazhagan N., Manikandan M., Sathishkumar M. (2018). Development of Pulsed Cold Metal Transfer and Gas Metal Arc Welding Techniques on High-Strength Aerospace-Grade AA7475-T761. J. Mater. Eng. Perform..

[2] Harender K., Neeraj K., Manmohan (2019). A Survey on Gas Metal Arc Welding (GMAW)—Review. Int. J. Sci. Res. Dev..

[3] Teixeira G.S., Mazzaferro J.A.E. (2019). GMA welding metal transfer mode study by high-speed imaging and electrical signal acquisition. J. Braz. Soc. Mech. Sci..

[4] Gubała S., Fidali M. (2014). Diagnostic method for welded joints based on the analysis of selected parameters of the welding process. Weld. Int..

[5] Feng S.Q., Hiroyuki O., Hidennori T., Yuichi K., Hu S.S. (2011). Qualitative and quantitative analysis of GMAW welding fault based on mahalanobis distance. Int. J. Precis. Eng. Manuf..

[6] Luksa K. (2006). Influence of weld imperfection on short circuit GMA welding arc stability. J. Mater. Process. Technol..

[7] Suban M., Tušek J. (2003). Methods for the determination of arc stability. J. Mater. Process. Technol..

[8] Zhang Z.F., Chen X.Z., Chen H.B., Zhong J.Y., Chen S.B. (2014). Online welding quality monitoring based on feature extraction of arc voltage signal. J. Mater. Process. Technol..

[9] Benaouda O.F., Mezaache M., Bouchakour M., Bendiabdellah A. (2023). Estimation of the droplet detachment frequency using SSAS and PSD techniques in GMAW process under different transfer modes. Int. J. Adv. Manuf. Technol..

[10] Sanchez W.D., Avila S.M., Brito J.V. (2022). A methodology based on empirical mode decomposition and synchrosqueezed wavelet transform for modal properties identification and damage detection. J. Braz. Soc. Mech. Sci..

[11] Kaur A., Ribton C., Balachandran W. (2016). Development of a novel approach for characterising electron beams and quality assurance of welds. J. Manuf. Process..

[12] Liu Y.H., Yang B., Han X.H., Tan C.W., Liu F.Y., Zeng Z., Chen B., Song X.G. (2022). Predicting laser penetration welding states of high-speed railway Al butt-lap joint based on EEMD-SVM. J. Mater. Res. Technol..

[13] He K.F., Si Y., Lu W., Lu Q.H., Li Q., Huang C.H., Xiao S.W. (2020). Time frequency feature extraction of the arc energy for quality detection of the aluminum alloy double pulse MIG welding. J. Adv. Mech. Des. Syst..

[14] Gong M.C., Kawahito Y., Li G., Gao M., Zeng X.Y. (2017). Stabilization effect of space constraint in narrow gap laser-arc hybrid welding analyzed by approximate entropy. Int. J. Adv. Manuf. Technol..

[15] Gu X.Y., Li H., Luo J.S. (2018). Lyapunov exponent analysis for the evaluation of hybrid laser double-arc welding process stability. Appl. Opt..

[16] Huang Y., Yang D.Q., Wang K.H., Wang L., Zhou Q. (2020). Stability analysis of GMAW based on multi-scale entropy and genetic optimized support vector machine. Measurement.

[17] Lv X.Q., Zhang R.X., Wang Y. (2016). Novel stability evaluation for short circuiting transfer based on features of input power. Sci. Technol. Weld. Join..

[18] Chen Y.T., Yang Z.D., Xu K., He P., Shi M.X., Chen S.J., Fang C.F. (2023). Effect of energy parameters on droplet transfer behavior and weld formation in laser-arc hybrid welding with cable-type welding wire. J. Mater. Res. Technol..

[19] Wen Y.M., Huang S.S., Xue J.X., Xie S.M. (2008). Observation and analysis of unstable metal transfer process in pulsed MIG welding. Trans. China Weld. Inst..

[20] Pincus S. (1995). Approximate entropy (ApEn) as a complexity measure. Chaos.

[21] Cao B., Xiang Y.P., Lv X.Q., Zeng M., Huang S.S. (2008). Approximate entropy—A new statistic to quantify arc and welding process stability in short-circuiting gas metal arc welding. Chin. Phys. B.

